# Supplementary material for: Exploration of T cell immune responses by expression of a dominant-negative SHP1 and SHP2
Source: Front Immunol. 2023 Jun 2;14:1119350. doi: 10.3389/fimmu.2023.1119350 (PMC10272835; doi:10.3389/fimmu.2023.1119350)
Supplement: Supplementary file 2 [file Table_1.docx]

| **Protein name** | **Peptide sequence** | **Peptide name** |
| --- | --- | --- |
| **PD1** | KEDPSAVPVFSVDYGELDFQWRE | ITIM |
|  | KEDPSAVPVFSVD**Y**GELDFQWRE | Phospho-ITIM |
|  | KTPEPPVPSVPEQTEYATIVFPSGMGTSS | ITSM |
|  | KTPEPPVPSVPEQTE**Y**ATIVFPSGMGTSS | Phospho-ITSM |
| **TIGIT** | LHDYFNVLSYRSLGNCSFFTETG | 1 |
|  | LHD**Y**FNVLS**Y**RSLGNCSFFTETG | p1 |
|  | LHD**Y**FNVLSYRSLGNCSFFTETG | p1.1 |
|  | LHDYFNVLS**Y**RSLGNCSFFTETG | p1.2 |
| **2B4** | TSPKEFLTIYEDVKDLKTRRNHE | 2 |
|  | TSPKEFLTI**Y**EDVKDLKTRRNHE | p2 |
|  | TFPGGGSTIYSMIQSQSSAPTSQ | 3 |
|  | TFPGGGSTI**Y**SMIQSQSSAPTSQ | p3 |
|  | TSQEPAYTLYSLIQPSRKSGSRK | 4 |
|  | TSQEPAYTL**Y**SLIQPSRKSGSRK | p4 |
|  | HSPSFNSTIYEVIGKSQPKAQNP | 5 |
|  | HSPSFNSTI**Y**EVIGKSQPKAQNP | p5 |
| **BTLA** | LEENKPGIVYASLNHSVIGPNSR | 6 |
|  | LEENKPGIV**Y**ASLNHSVIGPNSR | p6 |
|  | PNSRLARNVKEAPTEYASICVRS | 7 |
|  | PNSRLARNVKEAPTE**Y**ASICVRS | p7 |
| **PAG1** | QENMVEDCLYETVKEIKEVAAAA | 12 |
|  | QENMVEDCL**Y**ETVKEIKEVAAAA | p12 |
| **CTLA4** | KRSPLTTGVYVKMPPTEPECEKQ | 18 |
|  | KRSPLTTGV**Y**VKMPPTEPECEKQ | p18 |
|  | YVKMPPTEPECEKQFQPYFIPIN | 19 |
|  | **Y**VKMPPTEPECEKQFQP**Y**FIPIN | p19 |
|  | **Y**VKMPPTEPECEKQFQPYFIPIN | p19.1 |
|  | YVKMPPTEPECEKQFQP**Y**FIPIN | p19.2 |

**Supplementary Table 1:** List of peptide sequences derived from the endodomains of immune inhibitory receptors. Phosphorylated tyrosines are indicated in red.
